# (Sub-)Picosecond Surface Correlations of Femtosecond Laser Excited Al-Coated Multilayers Observed by Grazing-Incidence X-ray Scattering

**DOI:** 10.3390/nano14121050

**Published:** 2024-06-19

**Authors:** Lisa Randolph, Mohammadreza Banjafar, Toshinori Yabuuchi, Carsten Baehtz, Michael Bussmann, Nicholas P. Dover, Lingen Huang, Yuichi Inubushi, Gerhard Jakob, Mathias Kläui, Dmitriy Ksenzov, Mikako Makita, Kohei Miyanishi, Mamiko Nishiuchi, Özgül Öztürk, Michael Paulus, Alexander Pelka, Thomas R. Preston, Jan-Patrick Schwinkendorf, Keiichi Sueda, Tadashi Togashi, Thomas E. Cowan, Thomas Kluge, Christian Gutt, Motoaki Nakatsutsumi

**Affiliations:** 1 Department Physik, University of Siegen, 57072 Siegen, Germany; 2 European XFEL, 22869 Schenefeld, Germany; 3 Fakultät Physik, TU Dresden, 01069 Dresden, Germany; 4 Japan Synchrotron Radiation Research Institute (JASRI), Sayo 679-5198, Hyogo, Japan; 5 RIKEN SPring-8 Center, Sayo 679-5148, Hyogo, Japan; 6 Helmholtz-Zentrum Dresden-Rossendorf, 01328 Dresden, Germany; 7 Center for Advanced Systems Understanding (CASUS), 02826 Görlitz, Germany; 8 The John Adams Institute for Accelerator Science, Imperial College London, London SW7 2BW, UK; 9 Institute of Physics, Johannes Gutenberg-University, 55099 Mainz, Germany; 10 Kansai Photon Science Institute, National Institutes for Quantum Science and Technology, Kyoto 619-0215, Japan; 11 Fakultät Physik/DELTA, TU Dortmund, 44221 Dortmund, Germany

**Keywords:** grazing-incidence X-ray scattering, ultrafast surface dynamics, laser processing, XFEL

## Abstract

Femtosecond high-intensity laser pulses at intensities surpassing 10^14^ W/cm^2^ can generate a diverse range of functional surface nanostructures. Achieving precise control over the production of these functional structures necessitates a thorough understanding of the surface morphology dynamics with nanometer-scale spatial resolution and picosecond-scale temporal resolution. In this study, we show that single XFEL pulses can elucidate structural changes on surfaces induced by laser-generated plasmas using grazing-incidence small-angle X-ray scattering (GISAXS). Using aluminium-coated multilayer samples we distinguish between sub-picosecond (ps) surface morphology dynamics and subsequent multi-ps subsurface density dynamics with nanometer-depth sensitivity. The observed subsurface density dynamics serve to validate advanced simulation models representing matter under extreme conditions. Our findings promise to open new avenues for laser material-nanoprocessing and high-energy-density science.

## 1. Introduction

Intense, ultrashort laser–solid interactions at intensities ranging from 10^13^ to 10^16^ W/cm^2^ are of paramount importance for laser nanoprocessing to achieve functional surfaces. Among these interactions, one of the most notable examples is the generation of Laser-Induced Periodic Surface Structures (LIPSS) [1], which find applications, e.g., in antibacterial coatings, optical devices, chemical sensing, and tribology [2,3,4,5]. Understanding the dynamics of both surface and sub-surface phenomena at nanometer (nm) or even atomic scales, within the requisite temporal frame, is pivotal for comprehending the underlying physics responsible for creating desired surface structures in a controlled manner. Despite notable advancements in theoretical frameworks and models in recent years, aimed at elucidating intricate mechanisms involved in the self-organization of nanostructures under ultrashort laser irradiation within relevant temporal scales [6], direct experimental visualization of this process remains scarce. Thus far, the majority of experimental findings regarding surface manufacturing have relied on post-mortem analyses, which lack temporal dynamics information. Time-resolved experiments employing optical lasers, such as optical reflectometry, interferometry, and spectroscopy, suffer from limited spatial resolution and a lack of bulk sensitivity. Laser processing involves a complex chain of various physical processes occurring across different temporal and spatial scales. Initially, ultrashort laser interactions with metals excite electrons within the surface skin layer, typically spanning tens of nm. These excited electrons then propagate into the bulk at Fermi velocity before rapid thermalization occurs within sub-picoseconds (ps) timescales via collisions. Depending on the excitation strength and material properties, this process triggers electron-lattice/ion thermalization, coherent phonon oscillation, and lattice heating in ps timescales. This can lead to subsequent thermal or non-thermal melting, followed by ablation through spallation in sub-nanosecond(ns) timescales. These phenomena collectively contribute to later crystallization or amorphization, leading to specific surface nanostructures. Therefore, understanding above-mentioned early time phenomena in (sub-)ps dynamics is crucial. To address this need, here, we demonstrate experimental capabilities of visualizing physical processes at both surface and subsurface levels with ps and nm resolutions using an X-ray Free Electron Laser (XFEL) operating in a grazing-incidence geometry. Grazing-incidence X-ray small-angle scattering (GISAXS) is a well-established technique for probing lateral structures and correlations along the surface normal. It has been extensively utilized over the past decades at synchrotron X-ray facilities to characterize material structures across length scales ranging from sub-nm to micrometers (μm) [7,8], albeit limited to the millisecond timescale due to photon accumulation requirements.

Recently, we demonstrated the applicability of GISAXS to XFEL to track nanometric multilayer (ML) dynamics at unprecedented ps scales, which is six orders of magnitude faster than previously achievable [9]. This breakthrough is enabled by the XFEL’s ability to deliver an immense number of photons, comparable to those typically accumulated over a second using third-generation synchrotron X-rays, all within a single pulse lasting only ∼10 femtoseconds (fs). Despite inherent time smearing due to grazing-incidence, providing different parts of the X-ray arriving at the surface at different times, achieving approximately a ps time resolution is feasible.

Utilizing the in-plane scattering signal, characterized by the wavevector transfer along the sample depth ($Q_{z}$), which correlates closely with specular reflectivity [10], our experiments revealed the compressed, heated, intermixed, and ablated ML dynamics after the laser irradiation. On the other hand, out-of-plane diffuse scattering along the $Q_{y}$ direction provides insights into ultrafast changes in the lateral distribution of surface roughnesses. This can be described with the help of the height–height correlation function

(1) $$\begin{array}{c} C\left(R\right)\;=\;<h\left(0\right)h\left(R\right)>\;=\;\sigma^{2}\exp\left[-{\left(R/\xi_{\|}\right)}^{2H}\right] \end{array}$$

where *R* is the spatial separation of two points, σ is the root mean square (RMS) roughness, $\xi_{\|}$ is the lateral correlation length and *H* the Hurst parameter [11]. The correlation function along the sample depth can be written as

(2) $$\begin{array}{cc} <h_{j}\left(0\right)h_{k}\left(R\right)>\;=\; & \frac{1}{2}\left[\frac{\sigma_{k}}{\sigma_{j}}C_{j}\left(R\right)+\frac{\sigma_{j}}{\sigma_{k}}C_{k}\left(R\right)\right]\\ \; & \times \exp\left(-\frac{z_{j}-z_{k}}{\xi_{⊥}}\right). \end{array}$$

Here, C(R) is the height–height correlation function, *z* denotes the depth inside the sample and $\xi_{⊥}$ the cross-correlation length. The indices *j* and *k* denote different interfaces of the ML sample [12].

Additionally, by incorporating a low-Z capping layer on top of a high-Z ML, GISAXS facilitates measurements of energy transport several hundred nm within the material. Upon ultrashort laser irradiation, strongly confined temperature gradients induce compression (shock) waves, where the progression of temperature gradients depends on the mean-free-path of excited electrons which is a function of both temperature and density. Our results show that increasing laser excitation strength transitions the dominant energy transfer mode from compression wave dominance to the electron–thermal wave dominant regime.

## 2. Experimental Methods and Results

**Figure 1:**
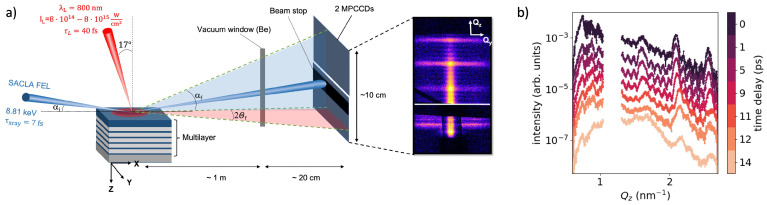
Schematics of the experimental setup to investigate the surface and subsurface solid-density plasma dynamics with GISAXS using single femtosecond X-ray FEL pulses. (**a**) An ML sample consists of 5 repetitions of tantalum (Ta) and copper nitride (Cu_3_N) of 4.5 and 8.5 nm thickness covered by a 200 nm aluminium capping layer. The samples were irradiated by an optical laser with a central wavelength of 800 nm, intensity of $(8\pm 0.2)\times 10^{14}$ or $\left(8\pm 0.2\right)\times 10^{15}\;W/cm^{2}$ with 40 fs pulse duration. The laser is irradiated by an incident angle of $17^{\circ }$ from the surface normal in *p* polarization. The X-ray pulses with 8.81 keV photon energy, 7 fs FWHM duration are irradiated on the sample at the grazing-incidence angle of $\alpha_{i}=0.75^{\circ }$, i.e., slightly above the critical angle of external total reflection of the layer materials. The laser beam is defocused to obtain a ∼500 μm spot diameter to cover the X-ray footprint of ∼300 μm at the surface. Scattered photons are recorded by 2 modules of the MPCCD area detector placed around the specular direction. The strong specular peak at $Q_{z}=1.16\;nm^{-1}$ is blocked. (**b**) In-plane signal along $Q_{z}$ (at $Q_{y}=0$) for different time delays between 0 and 14ps after the laser intensity peak.

The experiment was performed at the BL2 EH6 station at the SACLA XFEL facility in Japan [13]. A metallic ML sample, consisting of 5 repetitions of tantalum (Ta, 4.5 nm) and copper nitride (Cu_3_N, 8.5 nm) capped with a 200 nm thick Aluminum top layer was irradiated by an optical laser with a central wavelength of 800 nm, intensities of 8 × 10^14^ and 8 × 10^15^ W/cm^2^, pulse duration of 40 fs and a focus size of ∼500 μm (Figure 1a). After a variable delay time, the surface state of the sample was probed via surface-sensitive X-ray scattering employing ultrafast XFEL pulses of X-ray photons of energy 8.81 keV. The X-ray beam with a pulse energy of ∼0.1 mJ/pulse was incident under a grazing incidence angle of 0.75deg and the diffusely scattered intensity was recorded by a multi-port CCD (MPCCD) area detector [14] with the intense specular peak (incident angle equals exit angle, $Q_{\mathrm{specular}}=1.16\;nm^{-1}$) being blocked by a beam stop. More details about the experimental setup can be found in the Appendix A. Figure 1a displays a typical single-pulse GISAXS pattern. The shallow angle-of-incidence, in combination with the beam size of 4 μm leads to a large footprint (∼300 μm) of the beam on the sample surface, limiting the temporal resolution of the detected X-ray signal to ∼1 ps.

Figure 1b illustrates the in-plane scattering signal ($Q_{y}=0$) as a function of $Q_{z}$ for various delay times after laser irradiation. Because of the peculiar geometry of GISAXS, the in-plane scattering signal contains momentum transfers with components in both the normal ($Q_{z}$) and surface-parallel ($Q_{x}$) directions. As a result, the scattering signal reflects vertical density correlations along the z-direction ($Q_{z}$) of the ML structure, roughness correlations along the surface plane ($Q_{x}$) direction, and cross-correlations between different interfaces. For clarity, only the $Q_{z}$ values are plotted here, as $Q_{x}\ll Q_{z}$. The in-plane signal at $Q_{z}>Q_{\mathrm{specular}}$ is closely associated with the specular reflectivity curve [10], which we characterized ex situ. The spacing of the intense Bragg-like peaks at $Q_{z}=1.6$, 2.1 and $2.6\;nm^{-1}$ corresponds to the typical length scale, i.e., 13 nm thickness of each Ta/Cu_3_N double layer. The Kiessig fringes [15], represented as smaller peaks between the Bragg peaks, are a fingerprint of the number of double-layer repeats in the sample. Upon laser irradiation, substantial changes in the in-plane scattering signal are observed during the first 12 ps. The number and intensity of Kiessig fringes remain relatively stable during the initial ∼9 ps after laser irradiation, thereafter exhibiting a gradual decrease, concomitant with a simultaneous broadening and reduction of the intense Bragg-like peaks. After 12 ps, the Kiessig fringes are nearly gone, leaving behind only a broad residual of the Bragg-like peaks.

**Figure 2:**
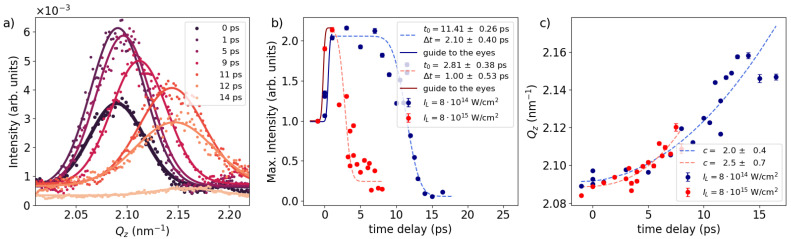
In-plane scattering signal along $Q_{z}$ around the Bragg-like peak at $2.1\;nm^{-1}$. (**a**) Circular dots represent the intensity of experimental data for various time delays, while solid lines correspond to a Gaussian model fit. (**b**) Peak intensity derived from the Gaussian model plotted against time delay. Circular dots denote the maximum intensity values from (**a**), while the dashed line represents a refinement using an error function. Blue color denotes the lower laser intensity of $8\times 10^{14}\;W/cm^{2}$ while red indicates the higher laser intensity of $8\times 10^{15}\;W/cm^{2}$. (**c**) $Q_{z}$-position of the Bragg-like peak. The dashed lines represent refinements using an exponential model.

The most notable structural changes inside the ML sample are manifested by the change of intensity and position of the intense peaks at $Q_{z}=1.6$, 2.1 and $2.6\;nm^{-1}$. Figure 2a illustrates the temporal evolution of the peak at $Q_{z}=2.1\;nm^{-1}$ following laser irradiation with an intensity of 8 × 10^14^ W/cm^2^. While only the peak at $Q_{z}=2.1\;nm^{-1}$ is displayed here, qualitatively similar behaviour is observed for the peaks at $Q_{z}=1.6$ and $2.6\;nm^{-1}$. The solid lines represent refinements using a Gaussian function. The peak amplitude as a function of time delay, extracted from the Gaussian refinement, is depicted in Figure 2b), with blue dots, which shows an ultrafast increase within the first ps. Subsequently, the intensity remains constant, followed by a gradual decrease at around ∼10 ps associated with a loss of structural correlation between the layers. This decay is modelled using an error function

(3) $$\mathrm{Intensity}=A\;\cdot \;\mathrm{erf}\left(-\frac{\left(x-t_{0}\right)}{\Delta t}\right)-B$$

resulting in values for $t_{0}=11.4\pm 0.3\;ps$ and a width of Δt=2.1±0.4 ps. Here, *A* represents a stretching factor, $t_{0}$ denotes the time at which the error function decreased to half of its initial value, Δt is the duration of the decrease, and *B* stands for a constant offset. In comparison, the same analysis for the higher-laser-intensity case of 8 × 10^15^ W/cm^2^, is depicted with red circular dots. Similar to the lower-intensity case, the ultrafast increase in scattering intensity within the first ∼1 ps is evident. However, for the time delays ≥1 ps the amplitude quickly begins to decline again. The refined error function yields a time constant of $t_{0}=2.8\pm 0.3\;ps$ and a width of Δt=1.0±0.5 ps. On top of the change in the Bragg intensity, we observe a steady increase in the Q-position with time delay for both laser intensities, as summarized in Figure 2c. This shift towards larger Q-values implies compression of the double-layer structure as the typical length scale decreases. Assuming that

(4) $$Q_{z}∼\mathrm{time}\;\mathrm{delay}{\mathrm{delay}}^{c}$$

we result in an exponent c=2.0±0.4 for the lower intensity and c=2.5±0.7 for the higher intensity, respectively.

**Figure 3:**
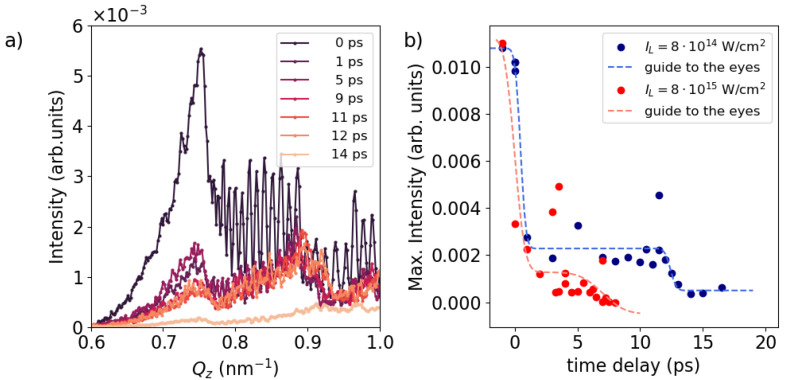
In-plane signal along $Q_{z}$ around the dynamical diffraction area between 0.6 and $1.0\;nm^{-1}$. (**a**) In-plane signal for different time delays at lower laser intensity case ($8\times 10^{14}\;W/cm^{2}$). (**b**) The circular dots display the integrated signal between $Q_{z}$ = 0.65–0.79 nm^−1^ as a function of time delay. The dashed line represents a guide to the eyes. Blue color stands for the lower laser intensity of $8\times 10^{14}\;W/cm^{2}$ and red denote the higher laser intensity $8\times 10^{15}\;W/cm^{2}$.

The dynamic diffraction effects at $Q_{z}<Q_{\mathrm{specular}}$ yield insight into the dynamics of the top Al layer. In particular, the scattering signals around exit angles equal to the critical angle for the total external reflection—$Q_{z}$ = $0.75\;nm^{-1}$ for Al—are particularly surface-sensitive, originating from an evanescent X-ray wave travelling parallel to the surface. This so-called Yoneda peak [16], primarily originated from interference within the topmost surface layer, serves as a sensitive marker of its surface structure. A close-up of this Yoneda peak is summarized in Figure 3a. The peak at $Q_{z}=0.75\;nm^{-1}$ exhibits a progressive decrease after laser irradiation, which persists for at least at t=12 ps before almost disappearing at t=14 ps, indicative of the ongoing presence of the solid density Al cover layer. The surface ablation speed can be approximated by the speed of sound, $C_{s}=\sqrt{\left(\gamma_{e}Z_{mean}k_{B}T_{e}+\gamma_{i}k_{B}T_{i}\right)/M_{i}}$, where $T_{e}$ and $T_{i}$ are electron and ion temperature, respectively, $Z_{mean}$ is the mean ionization, $\gamma_{e}∼1$ and $\gamma_{i}∼3$ are adiabatic index of electrons and ions, respectively and $M_{i}=26$ a.u. is the ion mass for Al [17]. In our temperature range ($T_{e}$ = 5–10 eV, $T_{i}$ = 1–5 eV) the ablation of the 200 nm layer takes 10–20 ps, which aligns with the timescale for the disappearance of scattering from Al observed in Figure 3a. The integrated intensity of the Yoneda peak ($Q_{z}$ = 0.65–0.79 nm^−1^) is depicted in Figure 3b for both low- (blue) and high- (red) intensity cases. In both cases, a rapid initial decay in intensity is observed within the first ps, followed by a period of quasi-constant signal intensity extended up to ∼12 ps and ∼5 ps before subsequent decrease, respectively. This temporal behaviour aligns qualitatively with that observed at the Bragg-like peak at higher $Q_{z}$ shown in Figure 2b. In Figure 3a, two additional Yoneda peaks are also visible at higher $Q_{z}$, corresponding to the Yoneda peaks for ${\mathrm{Cu}}_{3}N$ ($Q_{z}=0.86\;nm^{-1}$) and Ta ($0.95\;nm^{-1}$), respectively. Given that these materials are embedded within a 200 nm thick Al layer, the time-dependency of their intensity is not as pronounced as that of the Al Yoneda peak, except during the period between 12 and 14 ps when all peaks suddenly disappear. As we will discuss below, aided by plasma simulations, this phenomenon can be attributed to the arrival of a compression wave to the ML, which initiates significant modulation of the ML structure. An additional noteworthy feature in the figure is the presence of high-frequency fringes superimposed on the Ta and Cu_3_N Yoneda regions. These fringes arise from the interference between waves scattered from the Al surface and those from the Al-ML interface. Following laser excitation and the subsequent expansion of the top Al surface, these fringes rapidly vanish as a result of blurring of the distinct interface between vacuum and Al.

Both Bragg-like peaks and the Yoneda peak indicate the presence of three distinct time regimes. Immediately after laser irradiation ∼1 ps, we observe an ultrafast increase in the intensity of the Bragg-like peaks without any change in their $Q_{z}$-positions, accompanied by the decay of the Yoneda peak. As we will discuss further below, this phenomenon is attributed to a modification in the surface structural properties. In the second time frame, spanning from 1 to ∼10 ps or to ∼2 ps for low and high laser intensities, respectively, the intensities of the Bragg-like peaks and Yoneda peak remain constant, while the $Q_{z}$ position of the Bragg-like peaks steadily increases. This suggests that the embedded ML undergoes compression, although ML structure remains constant. In the third time frame, the intensities of the Bragg and Yoneda peaks are reduced, accompanied by a continued shift of the $Q_{z}$-position to even higher values. This indicates further compression of the entire ML, as well as significant modulation of the ML structure, resulting in the loss of correlation and subsequent decrease in the X-ray scattering intensity.

## 3. Discussion

**Figure 4:**
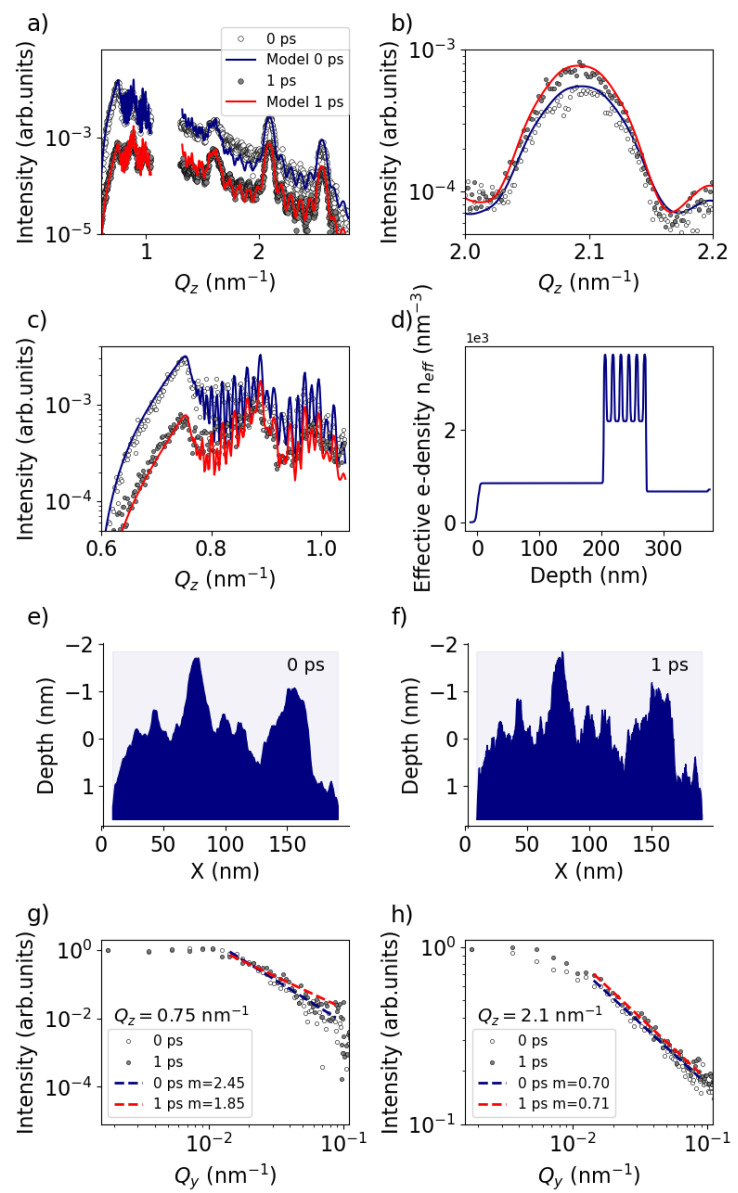
GISAXS signals and corresponding retrieved real-space electron density profile. (**a**) Lineout at 0 ps (white circular dots) and 1 ps (grey circular dots) after the laser intensity peak. Solid lines represent models using the program BornAgain (https://bornagainproject.org/). (**b**) Zoom into the area of the Bragg-like peak at $Q_{z}=2.1\;nm^{-1}$ and (**c**) zoom into the area of dynamical diffraction around $Q_{z}=0.75\;nm^{-1}$. (**d**) Retrieved real-space effective electron density profile as a function of the depth (Z). The laser irradiates the sample from the left. (**e**) Generated surface model for H=0.6, σ=2.3 nm and (**f**) H=0.15, σ=2.6 nm. (**g**) Lineout along $Q_{y}$ at the Al Yoneda peak ($Q_{z}=0.75\;nm^{-1}$). Dashed lines indicate a fit of the decay. (**h**) Lineout along $Q_{y}$ at $Q_{z}=2.1\;nm^{-1}$.

To facilitate a more quantitative discussion of the ultrafast surface dynamics, we employed the BornAgain [18] GISAXS analysis program (v 1.19) to model our experimental observations. The white circular dots in Figure 4a represent the experimental data at 0 ps while the grey dots depict the signal at a delay of 1 ps. The solid lines represent the BornAgain model corresponding to the effective electron density profiles shown in Figure 4d. Figure 4b,c provide enlarged views of the intense peak at $2.1\;nm^{-1}$ and the surface-sensitive Yoneda region, respectively. According to the model, the observed variation between the blue (0 ps) and red (1 ps) lines in Figure 4a–c can be attributed to the decrease in the Hurst parameter of the Al surface layer from 0.6 to 0.15, indicating an increase in the spatial frequency of roughness, as illustrated schematically in Figure 4e,f. This observation is supported by analyzing the out-of-plane signal along $Q_{y}$ at different $Q_{z}$ positions. Figure 4g illustrates a lineout along $Q_{y}$ at the Al Yoneda peak. The decay starting at $Q_{y}=0.01\;nm^{-1}$ is modeled via

(5) $$\mathrm{Intensity}∼Q_{y}^{-m}$$

where *m* defines the slope of the decay which is proportional to the Hurst parameter *H* [19]. Refining the lineouts for 0 and 1 ps indeed reveals a reduction of the Hurst parameter as *m* has decreased. Conversely, the lineout along $Q_{y}$ at larger $Q_{z}=2.1\;nm^{-1}$ (Figure 4h) shows no change in the Hurst parameter, indicating that the ultrafast change is localized to the surface rather than at the ML interfaces. Furthermore, it appears that in order to align the model with the experimental observation from 0 to 1 ps, the vertical correlation length needs to be increased from 100 to 150 nm, and the surface roughness RMS σ should be slightly increased from 2.3 to 2.6 nm. We hypothesize that this ultrafast change in surface roughness properties to be attributed to the presence of a thin aluminium oxide layer on the surface, which typically forms within minutes when exposed to air [20]. This oxide layer likely possesses a distinct surface morphology compared to the intrinsic surface morphology parameters of bare aluminium, leading to a reduction in spatial frequency of roughness as well as a decrease in vertical correlation length. Given that this layer is typically extremely thin, in the order of a nm, it evaporates instantaneously (<1 ps) after laser excitation revealing the intrinsic surface properties of the initial Al layer.

To substantiate our experimental findings further, we also conducted two advanced simulations: a 1D MULTI-fs hydrodynamic simulation and 1D PICLS Particle-in-Cell (PIC) simulation. The 1D MULTI-fs hydrodynamic simulation code is tailored for modeling short pulse high-intensity laser–solid interactions which directly solves the Maxwell equations. The code incorporates a temperature-dependent collision frequency, thermal conductivity ranging from metallic solids to high-temperature ideal plasmas, and a distinct equation-of-state (EOS) for electrons and ions (two-temperature model). The details of the simulation setups are summarized in Appendix A section. The 1D PICLS simulation incorporates the collisional 1d3v (one-dimensional in space and three-dimensional in velocity) [21]. An interpolated collision frequency akin to the MULTI-fs method was integrated into the code to address Angstrom-scale collisions occurring at electron temperatures ($T_{e}$) around the Fermi temperature ($T_{F}$).

**Figure 5:**
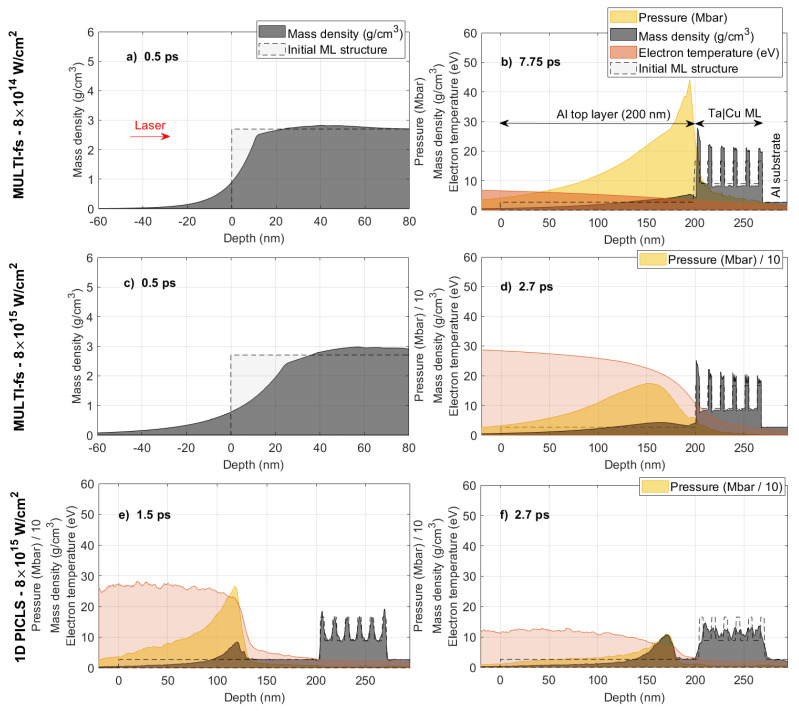
MULTI-fs hydrodynamic simulations irradiated by a p-polarized laser pulse with a duration of 50 fs, a wavelength of 800 nm, and a laser intensity of (**a**,**b**) $8\times 10^{14}\;W/cm^{2}$ and (**c**,**d**) $8\times 10^{15}\;W/cm^{2}$, respectively. Time delays are (**a**,**c**) 0.5 ps, (**b**) 7.75 ps and (**d**) 2.7 ps, respectively. The ML structure is the same as the experiment. (**e**,**f**) 1D Particle-in-cell PICLS simulation results at time delays of (**e**) 1.5 and (**f**) 2.7 ps, respectively. In all figures, the yellow, grey, and red regions represent the laser-induced pressure wave (in Mbar or Mbar/10), mass density (in $g/{\mathrm{cm}}^{3}$), and electron temperature (in eV), respectively. The dashed line denotes the initial sample density. The red arrow in (**a**) indicates the direction of the incoming laser.

Figure 5a,c display the density profile simulated using MULTI-fs at a delay of 0.5 ps. The localized heating induced by the laser within the skin depth instantaneously elevates the surface electron temperature to a few tens of eV and generates pressures exceeding >10 Mbar, prompting immediate surface expansion. This observation is aligned with the experiment demonstrating ultrafast changes in surface properties.

During the second time frame, spanning from 1 to ∼9 ps in the case of lower laser intensity ($I_{L}=8\times 10^{14}\;W/cm^{2}$), the intensity of the Bragg-like peak remains constant (Figure 2b). As confirmed by MULTI-fs simulations, it appears that between 1 and 8 ps the compression wave is only in the Al layer (Figure 5b), highlighted in yellow). Primarily, only the Al layer undergoes modulation during this interval, while the ML remains static with slight compression. Subsequently, after ∼8 ps, the simulation reveals a strong pressure wave compressing the ML. This aligns with the experimental findings (Figure 2c) that demonstrated a further increase in the $Q_{z}$-position of the Bragg peaks.

Figure 5c,d are the same simulation but performed at the higher laser intensity $I_{L}=8\times 10^{15}\;W/cm^{2}$. Here, the pressure peak arrives at the ML surface already after ∼3 ps, which encounters strong modulation subsequently. On the contrary, the experiment (Figure 2b) shows the significant decrease in Bragg intensity already started at ≤2 ps. It implies that the significant modulation of the ML occurs before the arrival of the strong compression wave to the ML surface, which is not seen in the MULTI-fs simulation.

It is known that Lagrangian hydrodynamic simulations, such as MULTI-fs, are incapable of accurately simulating the atomic mixing of adjacent layers, which inhibits particle interdiffusion between Lagrangian cells used in the code [9]. Given the ion thermal velocity ($\sqrt{k_{B}T_{i}/M_{i}}\geq$ 1 nm/ps at ∼1 eV thermal temperature, where $k_{B}$ is the Boltzmann constant, $T_{i}$ is the ion temperature and $M_{i}$ is the ion mass), particles are expected to penetrate into adjacent layers over timescales of several ps. To address this, we conducted 1D-PICLS kinetic simulation for the $I_{L}=8\times 10^{15}\;W/cm^{2}$ case, summarized in Figure 5e,f. While the simulation requires significantly higher computational cost, it properly includes the aforementioned kinetic effects. The simulation shows that the embedded ML undergoes significant layer intermixing from a thermal wave, even before the arrival of the strong compression wave. This phenomenon begins already at 1.5 ps delay as shown in Figure 5e. Even though the pressure peak is still in the middle of the Al layer, the ML has already started to undergo modulation. This leads to reductions in the Bragg peak in the X-ray scattering. By 2.7 ps delay, when both MULTI-fs and PICLS indicate the arrival of the pressure peak (Figure 5f), PICLS demonstrates that the entire ML is intermixed, and the original ML structure is entirely disrupted. Such a structure cannot produce any pronounced Bragg-like peaks due to the absence of a statistically relevant periodic structure. Note that the cooling is overestimated in PICLS (compare Figure 5d,f, highlighted in the yellow-shaded area) due to the absence of an opacity package in the code, which reabsorbs Bremsstrahlung radiation, as discussed in [9]. This results in reduced particle kinetic energy and is likely responsible for the density peaks observed at approximately 170 nm inside the Al layer in Figure 5f.

In summary, for the low-intensity case $I_{L}=8\times 10^{14}\;W/cm^{2}$, modifications to the ML structure are primarily driven by the compression wave, as the electron temperature is low enough that the thermal wave cannot significantly alter the ML before the arrival of the pressure wave. Conversely, for the high-intensity case $I_{L}=8\times 10^{15}\;W/cm^{2}$, significant heating occurs deep within the ML much before the arrival of the pressure wave.

## 4. Conclusions

In conclusion, our investigation of surface and subsurface dynamics in high-intensity laser-excited multi-layer (ML) systems using ultrafast grazing-incidence small-angle X-ray scattering (GISAXS) has provided important insights into the complex physical processes occurring in laser-matter interactions on a picosecond (ps) time scale.

By analyzing the GISAXS pattern, we distinguish between ultrafast sub-ps surface dynamics and subsequent multi-ps subsurface dynamics. The ultrafast modifications in surface properties, induced by strong localized surface heating, are evident in Yoneda region at low Q, where the scattering exit angle is close to the external total reflection. In contrast, the slower subsurface dynamics manifest as changes in intensity and position of the intense Bragg-like peak at $Q_{z}=2.1\;nm^{-1}$. Comparison of the temporal evolution of the density dynamics with 1D hydrodynamic and 1D particle-in-cell simulations reveals qualitative agreement across different time regimes observed in the experiment. Notably, the observed time scales align well with experimental observations, indicating the arrival of the pressure wave at the ML surface embedded in the 200 nm thick aluminium within a range of 3 to 10 ps and the intermixing of individual layers.

The presented GISAXS experiment was limited in time resolution to ∼1.2 ps due to the large footprint in grazing-incidence. However, this can be improved by using a smaller X-ray focal spot, e.g., 100 nm, which is readily available at XFEL facilities. This would imply only ∼30 fs smearing at 0.75° grazing-incidence, allowing experimental studies on surface and subsurface dynamics with nm spatial and femtosecond temporal resolution to observe the ultrafast changes on the surface or using laser intensities above 10^16^ W/cm^2^ to study, e.g., the effect of density oscillations [22]. 

## Data Availability

The data are available upon reasonable request.

## Appendix A

### Appendix A.1. Experimental Setup Details, X-ray and Laser Parameters, Timing Synchronization

The experiment was performed at the SACLA XFEL facility in Japan at the Experimental Hutch 6, which provides a high-intensity optical laser ($\lambda_{L}=800\;nm$ central wavelength, maximum 10 J/pulse with 40 fs duration in full-width half-maximum (FWHM)) combined with ultrashort intense X-ray pulses [13]. The X-ray pulses had a photon energy of 8.81 keV (with 43 eV FWHM bandwidth), ∼100 μJ/pulse, and a pulse duration of 7 fs in FWHM. The X-rays were focused to a 4 μm FWHM spot on sample by a set of compound refractive lenses placed 3 m upstream from the sample. The scattered X-ray signal was recorded on an MPCCD area detector with 50 μm pixel size [14] placed at a distance of 1.24 m from the sample and shielded by a 50 μm thick Al foil, to remove plasma-induced bremsstrahlung background. The incident angle was fixed at 0.75° to be slightly larger than the critical angle of total external reflection $\alpha_{c}\propto \sqrt{n_{e}}$ for all materials, Ta ($\alpha_{c\_\mathrm{Ta}}=0.46^{\circ }$), Cu ($\alpha_{c\_\mathrm{Cu}}=0.35^{\circ }$) and Al ($\alpha_{c\_\mathrm{Al}}=0.22^{\circ }$).

The samples were placed in a vacuum chamber and then irradiated by a high-intensity optical laser, impinging on the sample at $17^{\circ }$ incident angle from the surface normal with p-polarization. In order to cover the X-ray footprint on sample (4 μm FWHM for $0.75^{\circ }$ grazing incidence yields 300 μm), the optical laser beam was defocused to a diameter of ∼500 μm yielding an average laser intensity of about $8\times 10^{14}\;W/cm^{2}$ and $8\times 10^{15}\;W/cm^{2}$ depending on the attenuation. The synchronization between the X-ray and the laser was measured before starting of the experiment [13].

### Appendix A.2. Multi-fs

The 1D MULTI-fs hydrodynamic simulation code is tailored for modeling short (≤ps) pulse high-intensity laser-solid interactions at intensities below <10^17^ W$/{\mathrm{cm}}^{2}$ [23,24]. MULTI-fs directly solves the Maxwell equations, capturing ultrashort laser plasma dynamics with sharp plasma density gradients. The code incorporates a temperature-dependent collision frequency, thermal conductivity ranging from metallic solids to high-temperature ideal plasmas, and a distinct equation-of-state (EOS) for electrons and ions (two-temperature model). In the MULTI-fs simulation setup, the simulation box comprises 1478 cells distributed as follows: 400 cells for the 200 nm thick Al top layer, 678 cells for the ML, and 400 cells for a 200 nm thick Al substrate. To mitigate numerical artifacts, finer cell resolutions were employed near layer interfaces. We employed the EOS and ionization tables for aluminium (Al) as provided by the MULTI-fs package. For tantalum (Ta) and copper (Cu), the EOS and ionization data were generated using the FEOS code [25]. The radiation transfer module was disabled in the simulation, and the free streaming limiting factor was set to f=0.6. To maintain consistency with the experimental setup, the laser angle of incidence was fixed at $17^{\circ }$ from the sample normal. In our simulations, Al was chosen as the substrate material instead of silicon (Si) used in the experiment. This choice was motivated by well-established EOS of aluminium. Given that the energy transfer occurs from the ML to the substrate, we anticipate that the choice between these two materials will have minimal impact on the dynamics of the ML.

### Appendix A.3. 1D PICLS

The 1D PICLS simulation incorporates the collisional 1d3v (one-dimensional in space and three-dimensional in velocity) [21]. An interpolated collision frequency akin to the MULTI-fs method was integrated into the code to address Angstrom-scale collisions occurring at electron temperatures ($T_{e}$) around the Fermi temperature ($T_{F}$). To accurately simulate the microscopic particle collisions at the atomic scale, the spatial resolution was configured with a cell size of Δx=2 Å corresponding to a time–step of $\Delta t=6.67\times 10^{-19}$ s. Each computational cell accommodated 30 virtual ion particles with initial charge states of 2, 1, and 3 for Ta, Cu, and Al, respectively. Additionally, a fourth-order particle shape was employed, along with distinct particle weightings for different ion species. The ion number densities are set to realistic values for Ta ($n_{\mathrm{Ta}}=31.8n_{c}$), Cu ($n_{\mathrm{Cu}}=48.7n_{c}$), and Al ($n_{\mathrm{Al}}=34.6n_{c}$), where $n_{c}=m_{e}\omega_{L}^{2}/\left(4\pi e^{2}\right)=1.742\times 10^{21}{\mathrm{cm}}^{-3}$ represents the critical plasma density at the laser wavelength of $\lambda_{L}=800$ nm. Here, $m_{e}$ and *e* denote the electron mass and charge, respectively, and $\omega_{L}$ is the laser angular frequency. Ionization dynamics are modeled employing field and direct-impact ionization models. The laser incident angle is set as normal to the surface, as oblique incidence is not supported in 1D PICLS. Our test simulations using the MULTI-fs have revealed a negligible difference, approximately 1%, in laser absorption between normal incidence and a $17^{\circ }$ angle of incidence.

## References

[1] Bonse J., Gräf S. (2020). Maxwell Meets Marangoni—A Review of Theories on Laser-Induced Periodic Surface Structures. Laser Photonics Rev..

[2] Lutey A.H., Gemini L., Romoli L., Lazzini G., Fuso F., Faucon M., Kling R. (2018). Towards laser-textured antibacterial surfaces. Sci. Rep..

[3] San-Blas A., Martinez-Calderon M., Buencuerpo J., Sanchez-Brea L.M., del Hoyo J., Gómez-Aranzadi M., Rodríguez A., Olaizola S.M. (2020). Femtosecond laser fabrication of LIPSS-based waveplates on metallic surfaces. Appl. Surf. Sci..

[4] Li C.Y., Duan S., Yi J., Wang C., Radjenovic P.M., Tian Z.Q., Li J.F. (2020). Real-time detection of single-molecule reaction by plasmon-enhanced spectroscopy. Sci. Adv..

[5] Bonse J., Kirner S.V., Griepentrog M., Spaltmann D., Krüger J. (2018). Femtosecond laser texturing of surfaces for tribological applications. Materials.

[6] Rudenko A., Abou-Saleh A., Pigeon F., Mauclair C., Garrelie F., Stoian R., Colombier J. (2020). High-frequency periodic patterns driven by non-radiative fields coupled with Marangoni convection instabilities on laser-excited metal surfaces. Acta Mater..

[7] Roth S.V. (2016). A deep look into the spray coating process in real-time—The crucial role of X-rays. J. Phys. Condens. Matter.

[8] Su B., Körstgens V., Yao Y., Magerl D., Song L., Metwalli E., Bernstorff S., Müller-Buschbaum P. (2017). Pore size control of block copolymer-templated sol–gel-synthesized titania films deposited via spray coating. J. Sol-Gel Sci. Technol..

[9] Randolph L., Banjafar M., Preston T.R., Yabuuchi T., Makita M., Dover N.P., Rödel C., Göde S., Inubushi Y., Jakob G. (2022). Nanoscale subsurface dynamics of solids upon high-intensity femtosecond laser irradiation observed by grazing-incidence X-ray scattering. Phys. Rev. Res..

[10] Holý V., Kuběna J., Ohlídal I., Lischka K., Plotz W. (1993). X-ray reflection from rough layered systems. Phys. Rev. B.

[11] Sinha S.K., Sirota E.B., Garoff S., Stanley H.B. (1988). X-ray and neutron scattering from rough surfaces. Phys. Rev. B.

[12] Schlomka J.P., Tolan M., Schwalowsky L., Seeck O.H., Stettner J., Press W. (1995). X-ray diffraction from Si/Ge layers: Diffuse scattering in the region of total external reflection. Phys. Rev. B.

[13] Yabuuchi T., Kon A., Inubushi Y., Togahi T., Sueda K., Itoga T., Nakajima K., Habara H., Kodama R., Tomizawa H. (2019). An experimental platform using high-power, high-intensity optical lasers with the hard X-ray free-electron laser at SACLA. J. Sync. Rad..

[14] Kameshima T., Ono S., Kudo T., Ozaki K., Kirihara Y., Kobayashi K., Inubushi Y., Yabashi M., Horigome T., Holland A. (2014). Development of an X-ray pixel detector with multi-port charge-coupled device for X-ray free-electron laser experiments. Rev. Sci. Instrum..

[15] Kiessig H. (1931). Untersuchungen zur Totalreflexion von Röntgenstrahlen. Ann. Phys..

[16] Yoneda Y. (1963). Anomalous Surface Reflection of X-rays. Phys. Rev..

[17] Drake R.P. (2018). High-Energy-Density Physics.

[18] Pospelov G., Van Herck W., Burle J., Carmona Loaiza J.M., Durniak C., Fisher J.M., Ganeva M., Yurov D., Wuttke J. (2020). *BornAgain*: Software for simulating and fitting grazing-incidence small-angle scattering. J. Appl. Crystallogr..

[19] Salditt T., Metzger T.H., Peisl J. (1994). Kinetic Roughness of Amorphous Multilayers Studied by Diffuse X-ray Scattering. Phys. Rev. Lett..

[20] Nguyen L., Hashimoto T., Zakharov D.N., Stach E.A., Rooney A.P., Berkels B., Thompson G.E., Haigh S.J., Burnett T.L. (2018). Atomic-Scale Insights into the Oxidation of Aluminum. ACS Appl. Mater. Interfaces.

[21] Sentoku Y., Kemp A. (2008). Numerical methods for particle simulations at extreme densities and temperatures: Weighted particles, relativistic collisions and reduced currents. J. Comput. Phys..

[22] Paschke-Bruehl F.L., Banjafar M., Garten M., Huang L., Marré B.E., Nakatsutsumi M., Randolph L., Cowan T.E., Schramm U., Kluge T. (2023). Heating in Multi-Layer Targets at ultra-high Intensity Laser Irradiation and the Impact of Density Oscillation. New J. Phys..

[23] Eidmann K., Meyer-ter Vehn J., Schlegel T., Hüller S. (2000). Hydrodynamic simulation of subpicosecond laser interaction with solid-density matter. Phys. Rev. E.

[24] Ramis R., Eidmann K., Meyer-ter Vehn J., Hüller S. (2012). MULTI-fs—A computer code for laser–plasma interaction in the femtosecond regime. Comput. Phys. Commun..

[25] Faik S., Tauschwitz A., Iosilevskiy I. (2018). The equation of state package FEOS for high energy density matter. Comput. Phys. Comm..

